# Influence of Increased TaNbV on the Microstructure, Mechanical Properties, and Energy Release Characteristics of High-Entropy Alloy HfZrTi(TaNbV)_x_

**DOI:** 10.3390/ma18204713

**Published:** 2025-10-14

**Authors:** Chong Chen, Yusong Ma, Manhui Wei, Xiqiang Gai, Yue Peng, Yanqi Mei, Xinglong Liu, Kaichuang Zhang, Jianbin Li

**Affiliations:** Military Chemical Defense Institute, Beijing 102200, China; sjddh220@163.com (C.C.);

**Keywords:** high-entropy alloys (HEAs), dynamic mechanical properties, energetic structural materials

## Abstract

In this study, we propose a novel energetic structural material, HfZrTi(TaNbV)_x_ (x = 0.1, 0.3, 0.5, 0.7, 0.9, Ta:Nb: V = 1:1:1), to improve the ductility and toughness of the HfZrTi high-entropy alloy (HEAs). The transformation of the single-phase Hexagonal Close-Packed (HCP) HfZrTi-based alloy into a Body-Centered Cubic (BCC) phase HfZrTiTaNbV alloy can be achieved by tuning the concentration of Group VB β-stabilizing elements. The proposed alloy combines the insensitivity and excellent mechanical strength of conventional inert alloys with the ability to react with air under high-velocity impact for energy release. The mechanical properties and energy release characteristics of HZTX_x_ (H = Hf, Z = Zr, T = Ti, X = TaNbV) at various strain rates are systematically investigated, and comprehensive microstructural characterization is performed, establishing a clear structure–property relationship. Under high-rate loading, the rapid oxidation of reactive elements, such as Hf and Zr, with atmospheric oxygen releases substantial chemical energy, which can be further enhanced by an adiabatic temperature rise, inducing local thermal softening through adiabatic shear bands. This study elucidates the connection between the deformation response mechanism of HZTX_x_ under dynamic loading and the microstructure, providing crucial insights for advancing the application of high-entropy alloys in energetic systems.

## 1. Introduction

High-Entropy Alloys (HEAs) represent a novel class of alloys composed of five or more elements in equimolar or near-equimolar ratios. The high mixing entropy and severe lattice distortion inherent to these alloys typically contribute to their favorable balance between strength and ductility [1,2,3,4,5,6,7,8]. In 2010, researchers from the U.S. Air Force Research Laboratory, Senkov et al. [9,10,11,12], pioneered developing novel high-strength equiatomic single-phase BCC high-entropy alloys WNbMoTa and WNbMoTaV, composed of Group VB and VIB refractory elements. Subsequently, Group IVB transition metals, other transition metals such as Cr and Al, and non-metallic elements (including C, B, O, N, and Si), were gradually incorporated into the BCC high-entropy alloy systems. This expansion significantly broadened the compositional and property design space for these alloys. The BCC high-entropy alloys developed to date primarily form single-phase BCC solid solutions or structures featuring precipitates such as B2 or Laves phases within a BCC matrix. These alloys typically exhibit exceptionally high strength at room and elevated temperatures, with high-temperature mechanical properties that rival or surpass conventional superalloys [1]. This has attracted extensive research interest globally, making it a significant focus in high-entropy alloy research [13,14]. Research on the properties of BCC high-entropy alloys encompasses their physical, chemical, and mechanical performance. These alloys typically exhibit outstanding high-temperature mechanical properties and resistance to high-temperature softening—often surpassing those of traditional Ni-based superalloys [13,15,16,17,18,19]. Moreover, under extreme conditions such as ultra-high temperatures and hypervelocity impact [20,21,22], high-entropy alloys (HEAs), particularly refractory high-entropy alloys (RHEAs), exhibit further enhanced properties owing to the diverse composition and unique microstructures, demonstrating great potential as advanced structural materials. Furthermore, the compositional flexibility of HEAs enables the incorporation of highly reactive elements (e.g., Ti, Zr), making them promising candidates for energetic structural materials [20]. In 2017, Zhang et al. [23] proposed the concept of high-entropy alloys (HEAs) as energetic structural materials (ESMs) and demonstrated their feasibility, which represents a novel class of materials capable of releasing chemical energy under shock loading [24,25]. Ren et al. [24] studied the compressive properties and energy release characteristics of TiZrNbV high-entropy alloy under shock loading. The quasi-static compressive yield strength of TiZrNbV is 786 MPa. Furthermore, Chen et al. [26] investigated the WFeNiMo high-entropy alloy; its quasi-static compressive yield strength is 965 MPa. During penetration of thin steel targets, fragments of the WFeNiMo high-entropy alloy exhibited more intense energy release than tungsten alloys, with significant chemical reactions triggered by adiabatic shear.

Research by Zhang et al. [23] demonstrated that HfZrTiTa_0.53_, the quasi-static yield strength, compressive strength, and fracture strain of HfZrTiTa_0.53_ alloys reach 786 MPa, 1314 MPa, and 13.5%, respectively. TiZrNbV [27] HEAs, and Ti-Zr-Ta medium-entropy alloys exhibit substantial energy release during high-velocity impact [2], showcasing remarkable impact-induced energy release characteristics. However, RHEAs generally exhibit limited strain hardening capacity and, consequently, poor uniform deformability [28,29,30], due to a single deformation mechanism such as planar slip of dislocations and insufficient dislocation interactions within their single BCC phase or BCC-based matrix [31]. The increasing strain rate exacerbates this situation. When deformed under adiabatic dynamic conditions, thermal effects cannot be neglected, and adiabatic shear banding tends to dominate, leading to rapid failure [32]. The inadequate ductility of RHEAs hinders their application as structural materials due to their limited load-bearing reliability and processability.

As mentioned above, most BCC high-entropy alloys exhibit poor ductility at room temperature. Investigations into their mechanical properties have predominantly relied on compression testing, with relatively few studies focusing on tensile properties. These studies have primarily concentrated on the HfZrTiTaNb alloy system. In this work, we fabricated a series of HZTX_x_ (H = Hf, Z = Zr, T = Ti, X = TaNbV). High-entropy alloys by tuning the content of Ta, Nb, and V in an HfZrTi-based matrix alloy. This approach aims to enhance the room-temperature ductility and deformability of BCC-structured high-entropy alloys, thereby providing a strategy for their application in energetic structural materials such as casings.

## 2. Materials and Methods

### 2.1. Alloy Preparation

The HZTX_x_ high-entropy alloy was synthesized via suspension melting (LiDe Equipment Technology Co., Ltd., Hangzhou, China). Initially, raw metal materials (purity = 99.95%) were weighed and ultrasonically cleaned (KQ-500DB Yijin New Material Technology Co., Ltd., Beijing, China). The inner wall of the chamber and the crucible were polished with sandpaper, cleaned with ethanol, and dried. The prepared alloy was loaded into the levitation melting furnace crucible with low-melting-point elements placed at the bottom and high-melting-point elements on top. The furnace door was closed, and the four sealing knobs were tightened. The chamber was first evacuated to a low vacuum of 5 Pa using a mechanical pump, followed by evacuation to a high vacuum of 5 × 10^−3^ Pa using a molecular pump. The chamber was purged three times with argon before being backfilled with argon to a negative pressure of −0.05 MPa. The current regulator was adjusted to 70 A, and the melting process was maintained for 2 min. The current control valve was then shut off, and after the alloy cooled, the above steps were repeated for remelting. This melting cycle was repeated five times. The sample was removed after the furnace cooled to room temperature.

### 2.2. Microstructure Characterization

The crystal structure of the HZTX_x_ high-entropy alloy was characterized by X-ray diffraction (XRD; D8 ADVANCE) (Bruker, Billerica, MA, USA) with Cu-Ka radiation, and the measured 2θ ranges from 0 to 90°. Microstructure and elemental distribution were analyzed using scanning electron microscopy (SEM; Zeiss Gemini 300, Zhongke Baice Testing Technology Co., Ltd., Beijing China) equipped with backscattered electron (BSE) imaging, energy-dispersive spectroscopy (EDS) and transmission electron microscopy (TEM FEI Talos F200X, Zhongke Baice Testing Technology Co., Ltd., Beijing China) Electron backscatter diffraction (EBSD, Zhongke Baice Testing Technology Co., Ltd., Beijing China) analysis was performed to characterize the crystal orientation, grain/phase boundaries, phase distribution, and strain in the high-entropy alloy.

### 2.3. Mechanical Properties

**Figure 1:**
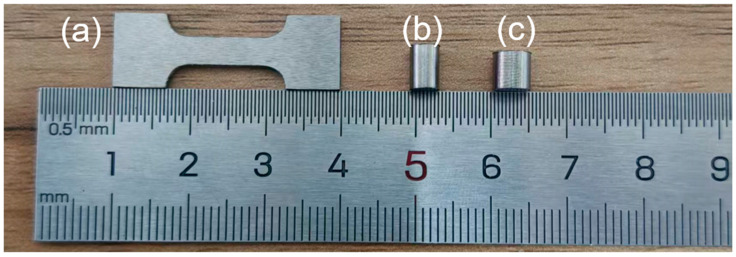
(**a**) Specimen subjected to quasi-static tensile testing, (**b**) Specimen subjected to quasi-static compressive testing, (**c**) Specimen tested via the split Hopkinson bar technique.

Figure 1a shows a dog-bone-shaped specimen subjected to quasi-static tensile testing, with standardized cross-sectional dimensions of 10 mm × 4 mm × 1.8 mm. (b) illustrates a cylindrical specimen with dimensions of Φ 4 mm × 6 mm (aspect ratio of 1.5:1) used for quasi-static compression testing. (c) depicts a cylindrical specimen machined to dimensions of Φ 5 mm × 5 mm for dynamic mechanical testing.

**Figure 2:**
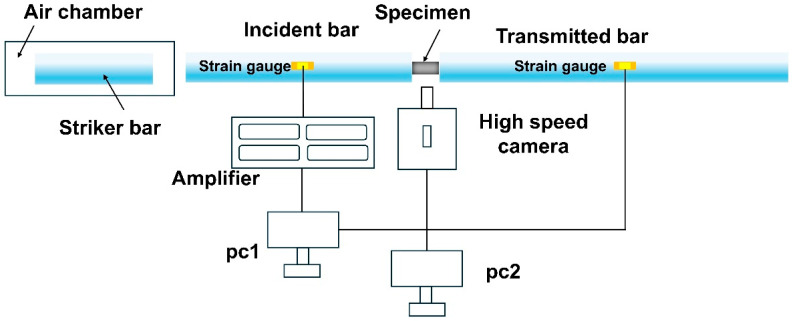
Split Hopkinson pressure bar apparatus.

Figure 2 shows a split Hopkinson pressure bar (SHPB) system (Longke Measurement and Control Technology Co., Ltd. Qinhuangdao, China), coupled with a high-speed camera was set up to investigate the dynamic compressive mechanical properties and fracture behavior of the material. The SHPB setup consisted of a striker bar (Φ20 mm × 400 mm), an incident bar (Φ20 mm × 1200 mm), and a transmission bar (Φ20 mm × 1200 mm), all made of 18NiCr350 maraging steel. Before conducting dynamic compression testing, strain gauges were mounted at the midpoints of the incident and transmission bars to record strain variations. Upon projectile impact with the incident bar, a high-speed camera was used to record the deformation process and fracture behavior of the specimen during dynamic loading.

## 3. Results and Discussion

### 3.1. Microstructure Characteristics

**Figure 3:**
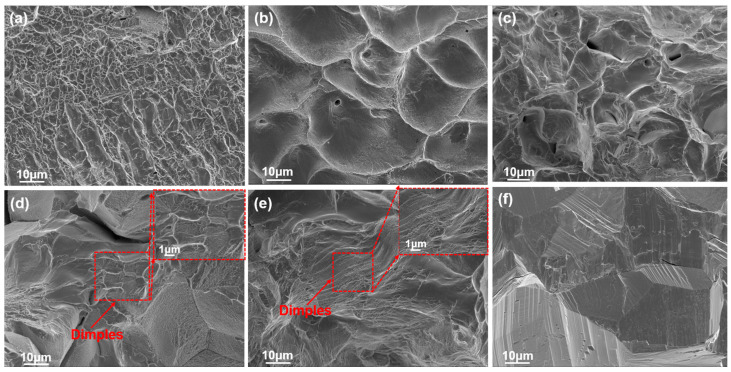
(**a**–**e**) SEM of the fracture surfaces of HZTX_0_, HZTX_0.3_, HZTX_0.5_, HZTX_0.7_, and HZTX_0.9_ specimens subjected to quasi-static tensile testing. The dashed boxes in (**d**,**e**) represent close-up views of the dimples. (**f**) SEM of the fracture surfaces of HZTX_0.1_ specimens subjected to quasi-static compression testing.

Figure 3a–e shows the quasi-static tensile fracture morphologies of the HZTX_0_, HZTX_0.3_, HZTX_0.5_, HZTX_0.7_, and HZTX_0.9_ alloy specimens. All fracture surfaces exhibit dimples that are either elongated or equiaxed, with the dimple size decreasing progressively as the content of X is increased. In contrast, the HZTX_0.1_ HEA demonstrates relatively poor ductility and is therefore represented solely by its quasi-static compressive fracture morphology. The fractographic features reveal cleavage planes in Figure 3f, thus indicating brittle fracture. These observations suggest that the content of X within the base alloy induces a transition from brittle to ductile fracture behavior.

**Figure 4:**
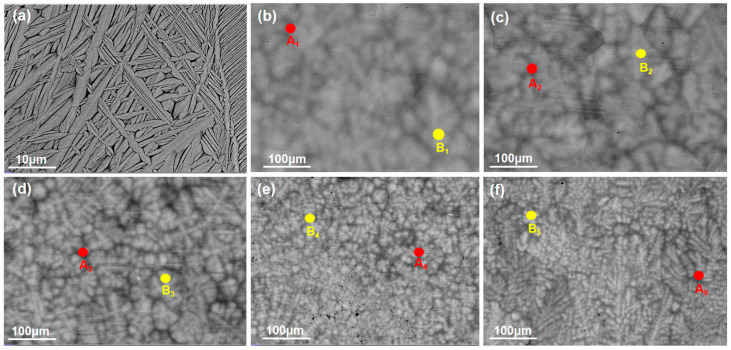
(**a**) BSE micrograph of the HZTX_0_, (**b**) BSE micrograph of the HZTX_0.1_, (**c**) BSE micrograph of the HZTX_0.3_, (**d**) BSE micrograph of the HZTX_0.5_, (**e**) BSE micrograph of the HZTX_0.7_, (**f**) BSE micrograph of the HZTX_0.9_. A_1_–A_5_: EDS point analysis locations on dendritic core; B_1_–B_5_: on interdendritic region.

**Figure 5:**
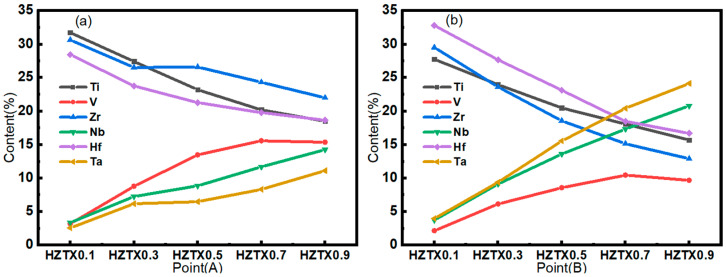
(**a**,**b**) Variations in the composition of A and B were determined by EDS.

The backscattered electron SEM image shown in Figure 4 reveals that the as-cast alloy exhibits equiaxed grains with a size of approximately 180 μm. The intragranular structure consists of a basket-weave morphology formed by interlaced elongated platelets with widths ranging from 0.1 to 1.5 μm. The basketweave structure is commonly observed in alpha titanium alloys. During the transformation from the high-temperature BCC phase to the low-temperature HCP phase, a single unit cell of BCC-Ti can give rise to 12 distinct HCP orientation variants. HCP lamellae nucleate and grow along these different orientations, resulting in the formation of an interlaced basketweave structure. As the concentration of Ta, Nb, and V increases, the HCP phase within the alloy completely disappears, leading to the formation of dark grain boundary segregation. The size of this segregation exhibits significant coarsening and a tendency to diffuse into the grain interior with increasing Ta, Nb, and V concentrations [33]. Meanwhile, Figure 5 shows EDS point scanning, performed on both the dendrites and interdendritic regions of the alloys with the addition of X to determine the distribution of each element. As the content of X is increased, the V element became progressively enriched in the interdendritic regions, while the Ta element was increasingly concentrated in the dendrite cores.

The phenomenon arises because the solidus temperature of the high-melting-point element Ta is first reached during the initial cooling of the molten alloy. As a result, the initially formed solid nuclei become preferentially incorporated into the developing dendritic cores, leading to significant Ta enrichment within these regions. As effective stabilizers of the BCC structure, increased concentrations of Ta, Nb, and V enhance the tendency toward single BCC phase formation. Specifically, higher Ta and Nb contents provide more high-melting-point solvent elements that solidify first to form the dendritic framework, while a greater amount of V, being a lower-melting-point solute, is rejected into the liquid phase. Consequently, the extent of compositional segregation intensifies with increasing total content of these elements, making the coexistence of V-enriched interdendritic regions and Ta-rich dendritic cores more pronounced.

**Figure 6:**
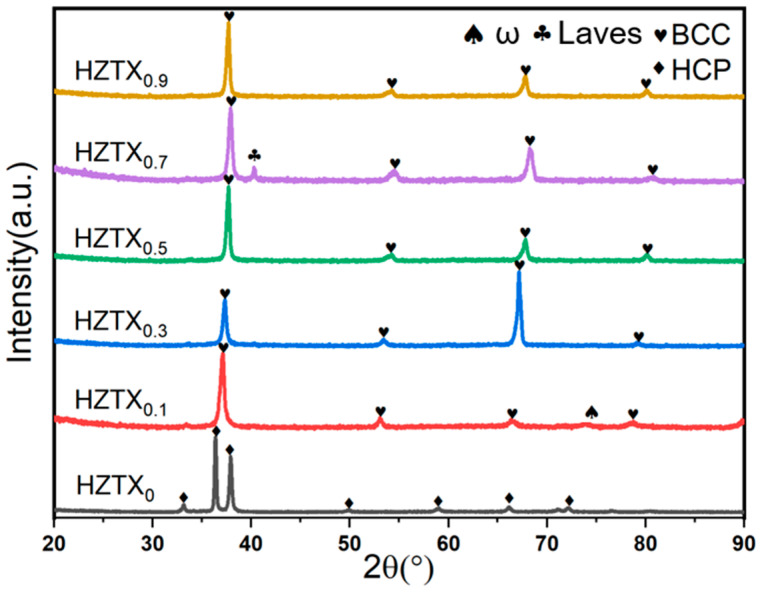
XRD patterns of HZTX_x_ HEAs.

**Figure 7:**
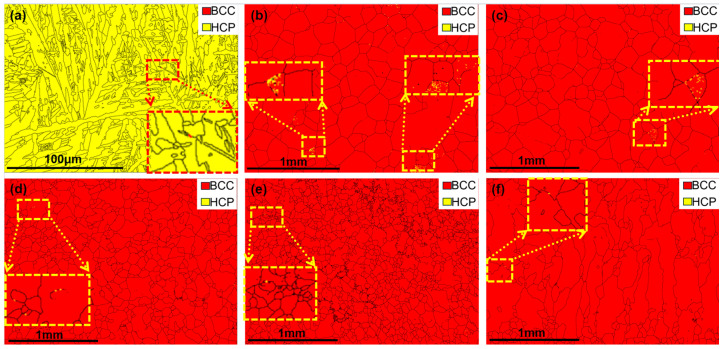
(**a**) EBSD phase maps of the HEAs of HZTX_0_, (**b**) EBSD phase maps of the HEAs of HZTX_0.1_, (**c**) EBSD phase maps of the HEAs of HZTX_0.3_, (**d**) EBSD phase maps of the HEAs of HZTX_0.5_, (**e**) EBSD phase maps of the HEAs of HZTX_0.7_, (**f**) EBSD phase maps of the HEAs of HZTX_0.9_. In the dashed boxes: close-up views of the secondary phase from EBSD analysis.

Figure 6 and Figure 7 show the phase composition and structural evolution of as-cast HZTX_x_ HEAs, which were characterized by XRD and EBSD analyses. The results reveal a targeted change in phase stability. Figure 6 and Figure 7 reveal a single hexagonal close-packed (HCP) phase structure in the HZTX_0_ high-entropy alloy, with its XRD pattern exhibiting exclusively HCP diffraction peaks. As the x content increases, the EBSD maps detect a predominant BCC phase along with a minor HCP phase. In contrast, the peak of the Laves phase in HZTX_0.7_ likely indicates the formation of HfV_2_. The limited volume fraction of the residual HCP phase, however, was not detected by XRD. It can be observed that with the increase in x content, the volume fraction of the BCC phase increases, indicating that the formation of the hcp phase is suppressed while the formation of the BCC phase is promoted. SAED further confirms that the crystal undergoes a phase transition from HCP to BCC with an increased content of X.

**Figure 8:**
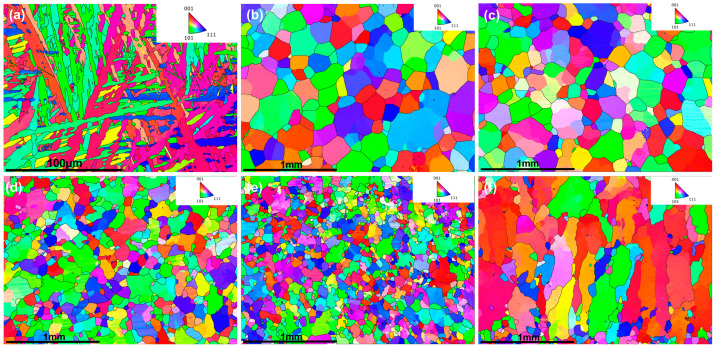
(**a**–**f**) presents the EBSD orientation maps for the HZTX_x_ high-entropy alloy system with the X content increasing from 0 to 0.9.

The EBSD analysis in Figure 8 shows that, as the X content increased from 0.1 to 0.7, the alloy grains progressively transitioned from a basket-weave morphology to irregular polygons with random orientations. However, HZTX_0.9_ exhibited a preferred ⟨100⟩ orientation. This is because the ⟨100⟩ direction in BCC-structured HEAs has the highest atomic linear density and lowest growth resistance, making it energetically favorable. Grains with ⟨100⟩ parallel to the heat flow thus grew fastest and outcompeted others [34]. The excessive addition of high-melting-point elements (Ta, Nb, V) elevated the liquidus temperature of the HEAs, thereby increasing the undercooling and the thermal energy required for dissipation during solidification. The resulting large undercooling and steep thermal gradients provided a stronger, sustained driving force for competitive ⟨100⟩ growth.

**Figure 9:**
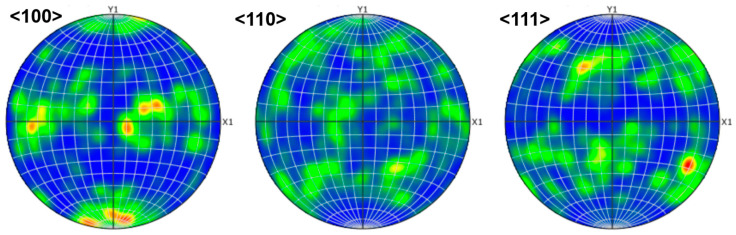
The pole figure of HZTX_0.9_ with <100>, <110>, <111> orientation.

The formation of equiaxed grains requires a key mechanism: constitutional supercooling [35]. This means that the enrichment of solute at the solidification front depresses the local melting point, thereby forming a Constitutional Supercooling Zone (CSZ), which creates conditions for new heterogeneous nucleation. Elements like Hf, Zr, and Ti have a pronounced segregation tendency during solidification. In contrast, Ta, Nb, and V (particularly Ta and Nb) have relatively lower diffusion coefficients in the BCC structure and exhibit good compatibility with the principal elements. Figure 9 shows the pole figure, which indicates a predominance of grains with the <100> orientation in agreement with the above conclusion.

**Figure 10:**
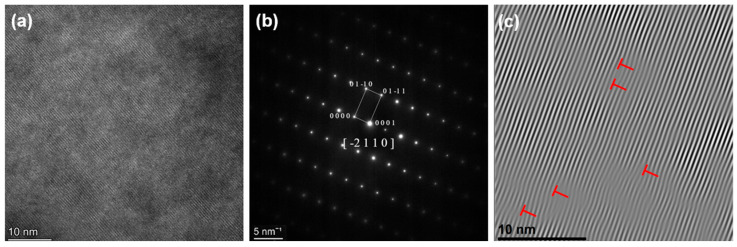
(**a**) HRTEM, (**b**) SAED, (**c**) IFFT of HZTX_0_.

**Figure 11:**
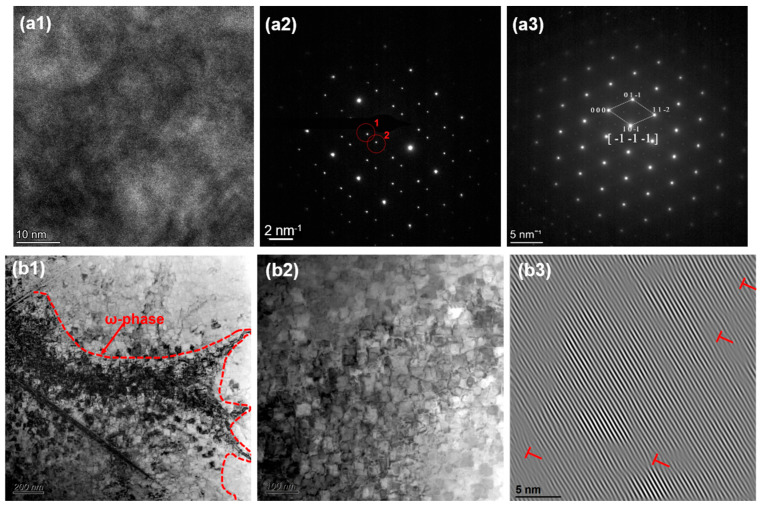
(**a1**) HRTEM, (**a2**,**a3**) SAED, (**b1**,**b2**) BF, (**b3**) IFFT of HZTX_0.1_.

As evidenced by SAED (Selected Area Electron Diffraction) in Figure 10 and Figure 11, an increase in the content of X was found to promote a phase transition in the high-entropy alloy from an HCP to a BCC structure, which is consistent with the results shown in Figure 5 and Figure 6.

The ω phase was observed in alloys with low Ta, Nb, and V contents, such as HZTX0.1. This is as evidenced by the diffraction spots at positions 1 and 2 circled in Figure 11(a2) and further confirmed by the bright-field (BF) images in Figure 11(b1,b2). Additionally, the XRD pattern of HZTX0.1 in Figure 6 shows diffraction peaks corresponding to the ω phase. This is attributed to the fact that Ti, Zr, and Hf are typical ω-phase-forming elements, which tend to undergo a diffusionless, displacive transformation from the β-phase [36]. The ω-phase, which has a hexagonal structure, is hard and brittle, and generally detrimental to ductility. In contrast, elements such as Ta, Nb, and V are strong β-stabilizers. They effectively lower the free energy of the β-phase (BCC structure) and expand the temperature range of the β-phase field, thereby stabilizing the β-phase over a wider composition and temperature range and suppressing the precipitation of other phases, such as the α- and ω-phases. When the content of these strong β-stabilizers is reduced, the average electron concentration (e/a) of the alloy system decreases [37]. A lower e/a ratio is directly associated with instability of the β-phase, making metastable phases such as the ω-phase thermodynamically more favorable and prone to precipitation. Furthermore, the formation of the ω-phase is a diffusionless transformation whose nucleation strongly depends on local soft-mode vibrations and lattice instabilities in the parent BCC phase. With a reduction in Ta, Nb, and V content, the composition becomes dominated by Ti, Zr, and Hf. The atomic size mismatch among these elements exacerbates local strain along specific crystallographic directions in the BCC lattice. This strain provides an ideal condition for the coordinated atomic displacement (collapse) required for ω-phase formation, thereby facilitating its nucleation.

**Figure 12:**
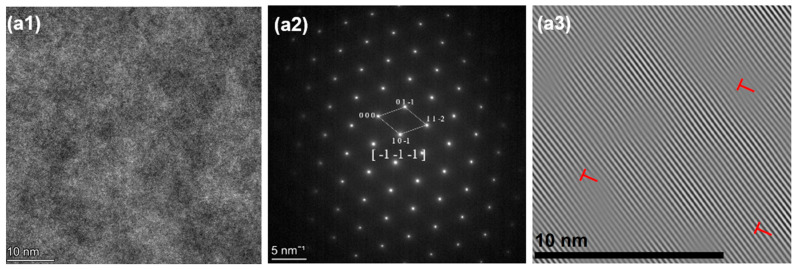

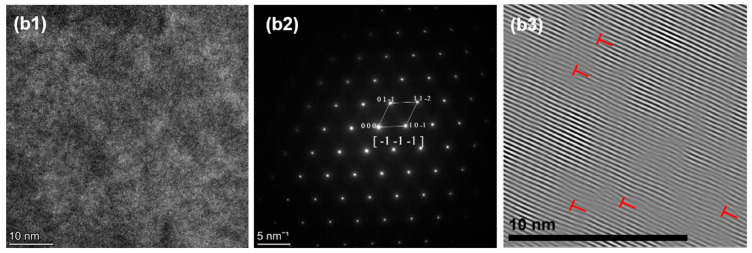
(**a1**) HRTEM, (**a2**) SAED, (**a3**) IFFT of HZTX_0.3._ (**b1**) HRTEM, (**b2**) SAED, (**b3**) IFFT of HZTX_0.5_.

**Figure 13:**
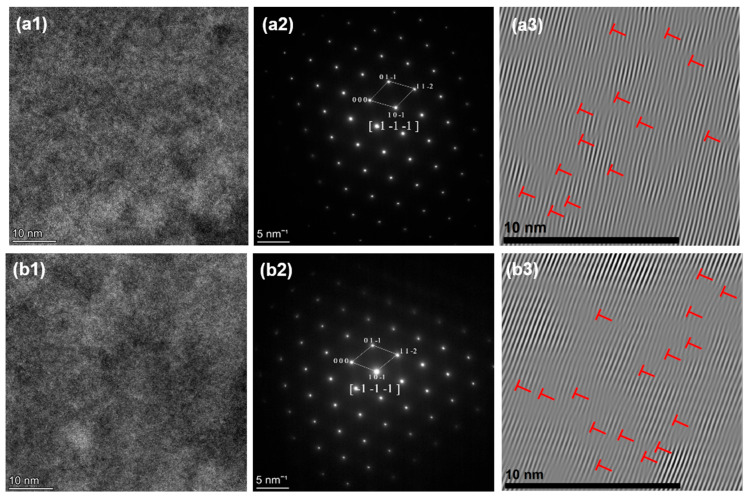
(**a1**) HRTEM, (**a2**) SAED, (**a3**) IFFT of HZTX_0.7._ (**b1**) HRTEM, (**b2**) SAED, (**b3**) IFFT of HZTX_0.9_.

Also shown in Figure 12(a3,b3) and Figure 13(a3,b3), the IFFT of HZTX_x_ reveals that the density of lattice distortion increases with increasing X content, which is attributed to the large atomic radius difference between Ta and V. The increased lattice distortion density significantly enhances the solid solution strengthening effect. The addition of Ta, Nb, and V alters the elastic modulus in local regions, requiring dislocations to expend additional energy to traverse these regions with varying elastic moduli. 

A strong interaction occurs between the internal stress field generated by the distortion and the stress field of the dislocations themselves, resulting in the pinning of dislocations and the hindrance of their slip, ultimately leading to an improvement in the material’s strength. Severe lattice distortion effectively strengthens the alloy while considerably restricting the mobility of dislocations. The initiation and propagation of dislocations become difficult, necessitating high stress during the initial stages of deformation. Once deformation begins, dislocations rapidly entangle and accumulate, impeding coordinated plastic deformation. 

**Figure 14:**
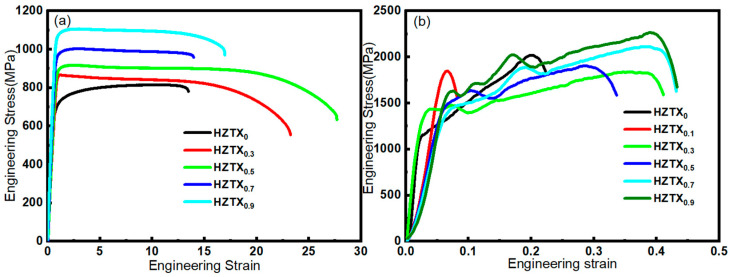
(**a**) Quasi-static tension stress–strain curve at a strain rate of 1 × 10^−3^s^−1^; (**b**) stress–strain curve at a strain rate of 4000 s^−1.^

The severe lattice distortion may cause a high work hardening rate initially, but dislocation multiplication and storage soon reach saturation, leading to a reduction in uniform plastic deformation capability. The elongation is typically reduced, which is consistent with the quasi-static tensile properties shown in Figure 14a.

### 3.2. Mechanical Properties of HZTX_x_

Figure 14 demonstrates the quasi-static tensile properties of X. The addition of TaNbV enhanced both the strength and ductility of the HEAs, except HZTX_0.1_, where the sample exhibited brittleness and poor tensile properties—consistent with the dynamic mechanical testing results. When the content of X exceeds 0.5, the strength of the high-entropy alloy increases while the elongation after fracture decreases. Notably, the elongation after fracture of HZTX_0.9_ shows an increase compared to that of HZTX_0.7._ The increase in plasticity may be attributed to grain growth resulting from the preferred orientation of grains. The yield strength, ultimate tensile strength, and elongation at fracture are summarized in Table 1.

The figure shows the engineering stress–strain curves of the HZTX_x_ at a strain rate of 4000 s^−1^. Except for the HZTX_0.1_ specimen, which exhibited brittle fracture, all other samples demonstrated appreciable plastic deformation. The strength of the alloy increased with higher TaNbV content. Meanwhile, the HEAs exhibit positive strain rate sensitivity under high strain rate conditions.

Firstly, the yield strength of the HEAs increased with rising TaNbV content, albeit at the expense of reduced elongation in HZTX_0.7_ and HZTX_0.9_. This strength-ductility trade-off originated from the severe lattice distortion induced by the large atomic radius mismatch of V. The distortion created effective barriers to dislocation motion, significantly increasing the resistance to glide and thereby elevating the yield strength.

Secondly, solute atoms (such as Ta, Nb, and V) engage in elastic interactions with dislocations in the matrix. Larger Ta and Nb atoms tend to aggregate in the compressive stress regions beneath dislocation lines, while smaller V atoms may accumulate in tensile stress regions, thereby “pinning” the dislocations. Additionally, the elastic moduli of these elements differ. Dislocations tend to propagate preferentially through regions with lower modules. The introduction of Ta, Nb, and other elements introduces modulus mismatches, which further hinder dislocation motion. The increased content of Ta, Nb, and V directly enhances this solid solution strengthening effect, making plastic deformation more difficult, thereby improving strength but reducing ductility.

Furthermore, Ta and Nb are strong carbide-forming elements with high melting points and inherent stability. Their addition is likely to enhance the interatomic bonding strength. Stronger atomic bonds require higher energy to break for dislocation glide to occur, which also contributes to the increased strength and decreased plasticity.

### 3.3. SHPB Test of HZTX_x_ Alloy

Based on the split Hopkinson pressure bar (SHPB) tests, the deformation and fracture process of the HEA-ESM specimen under dynamic loading in an air atmosphere was presented. The HEA-ESM specimen was sandwiched between the incident bar and the transmission bar. High-speed camera images from Hopkinson bar experiments at a strain rate of 4000 s^−1^ were selected for HZTX_0_, HZTX_0.1_, and HZTX_0.9_.

Figure 10 shows the adiabatic shear band formed in HZTX_0_ at t = 0.14 ms. Spark emission is observed at t=0.69 ms. This phenomenon is attributed to the following mechanism: upon application of a high-strain-rate load, homogeneous plastic deformation is initiated. The vast majority (typically over 90%) of the plastic work is converted into heat, resulting in adiabatic temperature rise. This effect is particularly pronounced in refractory high-entropy alloys such as HZT, which exhibit high strength and relatively low thermal conductivity. The local accumulation of heat causes a sharp temperature increase in specific regions. The rise in temperature leads to a decrease in flow stress, a phenomenon known as thermal softening. Consequently, the localized region becomes softer than the surrounding material. Spark formation is attributed to oxidation reactions between fragments and oxygen. During loading, heat is generated within the fragments due to adiabatic temperature rise caused by shear friction.

According to the principle of least resistance, further plastic deformation becomes concentrated preferentially within this softened zone. This concentration of deformation generates additional heat, leading to even higher temperatures and more significant thermal softening, which in turn further softens the region. As the material softens, deformation becomes increasingly localized, producing yet more heat. Instability is triggered when the rate of heat generation exceeds the capacity for heat dissipation, resulting in the formation of a highly localized shear band dominated by the thermal softening effect.

**Figure 15:**
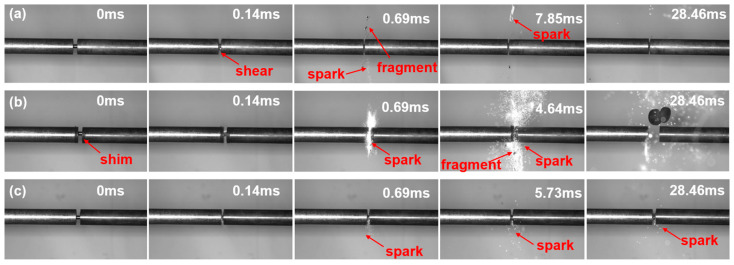
(**a**) The deformation and fracture process under Air atmospheres of HZTX_0_, (**b**) The deformation and fracture process under Air atmospheres of HZTX_0.1_, (**c**) The deformation and fracture process under Air atmospheres of HZTX_0.9_.

Due to the substantial precipitation of the ω-phase, the HZTX_0.1_ specimen exhibits low strength in static mechanical tests. In dynamic tests, this low strength leads to more complete fragmentation. To prevent severe oxidation and adhesion to the incident bar during Hopkinson bar tests resulting from extensive fragmentation, the specimen was clamped between spacers made of the same material as the incident and transmission bars, as shown in Figure 15b. Figure 15b displays intense sparking and fragment ejection observed in the HZTX_0.1_ specimen at t = 4.64 ms. Furthermore, active elements in the high-entropy alloy react with air under local high-temperature conditions, ultimately producing sparks.

**Figure 16:**
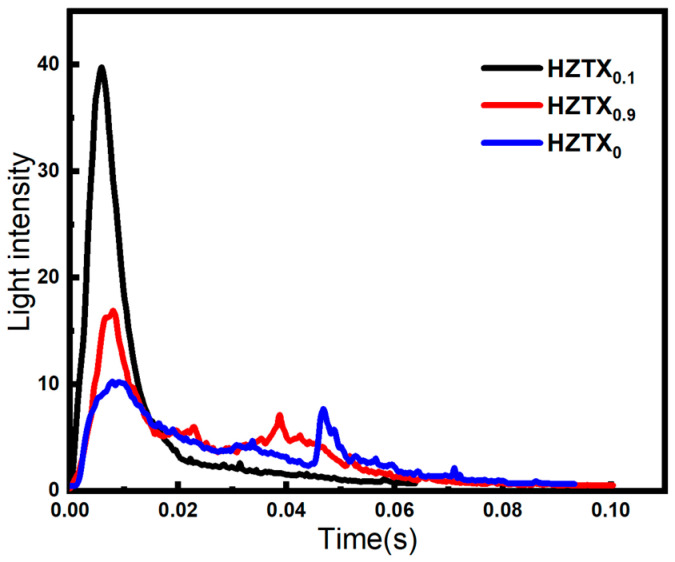
Time-Dependent Light Intensity Profiles of HZTX_0_, HZTX_0.1_, HZTX_0.9_.

The faster the time to maximum brightness upon specimen fragmentation and the greater the light intensity, the more efficient the energy release. To semi-quantify the energy release characteristics of the HEAs, the high-speed photographic images were binarized, with the threshold for optical radiation intensity set to 125–245. Pixels exhibiting grayscale values greater than 125 were identified as those corresponding to energy release reactions. Statistical analysis was performed on the luminous regions associated with energy release reactions in the high-speed images. As indicated in the Figure 16, HZTX_0.1_ exhibits the earliest ignition and the highest combustion luminosity. Its brittle characteristics facilitate more complete fragmentation, leading to a more violent oxidation reaction. HZTX_0.9_ ignites slightly earlier and shows higher luminosity than HZTX_0_. These results demonstrate that HZTX_0.9_, while satisfying the requirements of high strength and high plasticity, still maintains a specific energy release performance, making it a potential candidate for energetic liner materials.

## 4. Conclusions

In summary, this work developed an HfZrTiTaNbV(HEA) with excellent mechanical properties under both quasi-static and dynamic loading by tailoring the content of Ta, Nb, and V elements. The main findings are as follows:

(1) As the content of Ta, Nb, and V was increased, the HZTX_x_ high-entropy alloy underwent a transition in mechanical behavior from ductile to brittle and then back to ductile, accompanying by a phase evolution from a single hexagonal close-packed (HCP) structure to a body-centered cubic (BCC) phase with omega (ω)-phase precipitation, and ultimately to a single-phase BCC structure.
(2) Under quasi-static tension, the profuse precipitation of the ω phase in HZTX_0.1_ resulted in brittle fracture and low strength, leading to poor tensile properties. As the content of Ta, Nb, and V increased, the grain size was progressively reduced, and the resultant grain refinement endowed the other alloys with improved tensile performance. The concomitant lattice distortion further contributed to an enhancement in yield strength. HZTX_0.9_ exhibited a yield strength of 1065 MPa, an ultimate tensile strength of 1119 MPa, and a fracture elongation of 16.4%
(3) In the split-Hopkinson pressure bar (SHPB) tests, high-speed imaging revealed that the selected alloys exhibited energy-releasing oxidation reactions under dynamic high-strain-rate loading. The primary mechanism is attributed to the oxidation of active elements (e.g., Hf, Zr) with atmospheric oxygen during the fragmentation process, resulting in visible sparking. The brittle HZTX_0.1_ alloy fragments more thoroughly, producing finer fragments that facilitate more complete oxidation of the active elements. This results in the earliest observed ignition and the most intense sparking. Meanwhile, the HZTX_0.9_ alloy exhibits higher strength and superior ductility compared to the base alloy A, while also demonstrating a greater energy release effect. This combination of properties provides key material support for the future development of energetic structural materials.

## Figures and Tables

**Table 1:** The mechanical property parameters of the HZTX_x_ HEAs.

Category	Yield Strength (MPa)	Ultimate Tensile Strength (MPa)	Elongation at Fracture (%)
HZT	642	811	13.5%
HZTX_0.3_	830	869	22.0%
HZTX_0.5_	894	934	27.3%
HZTX_0.7_	963	1011	12.3%
HZTX_0.9_	1065	1119	16.4%

## Data Availability

The original contributions presented in this study are included in the article. Further inquiries can be directed to the corresponding authors.
